# Preventing Post-induction Hypotension During General Anaesthesia: A Dose-Response Study of Ringer’s Lactate Guided by the Inferior Vena Cava Collapsibility Index

**DOI:** 10.7759/cureus.110113

**Published:** 2026-06-02

**Authors:** Abhishek Chatterjee, Merina Sam, Himanshu Kumar, Deb Sanjay Nag, Bappaditya Pal, Aleena Wilson, Kakan Chakraborty

**Affiliations:** 1 Anaesthesiology, Tata Main Hospital, Jamshedpur, IND; 2 Paediatric Anaesthesia, King Edward Memorial Hospital and Seth G.S. Medical College, Mumbai, IND; 3 Anesthesiology, Tata Main Hospital, Jamshedpur, IND

**Keywords:** dose response study, general anesthesia, inferior vena cava collapsibility index, post-induction hypotension, ringer's lactate

## Abstract

Introduction

Post-induction hypotension (PIH) is a frequent and serious complication during general anesthesia (GA), leading to adverse outcomes like myocardial injury and acute kidney injury. Current methods for assessing preoperative volume status are often unreliable or invasive. Ultrasonography-guided Inferior Vena Cava Collapsibility Index (IVCCI) offers a non-invasive alternative for predicting fluid responsiveness. While IVCCI-guided fluid loading effectively prevents spinal anesthesia-induced hypotension, its role and the optimal Ringer's lactate (RL) dose in GA patients remain unclear. This study aimed to determine the optimal intravenous RL dose to prevent PIH, guided by IVCCI.

Methods

This prospective, randomized, interventional study enrolled 88 adult patients with American Society of Anesthesiologists (ASA) physical status I/II undergoing elective general surgery at Tata Main Hospital, Jamshedpur, India, from July 2023 to August 2024. Participants with an IVCCI ≥ 40% were randomized into two groups: Group A received 10 ml/kg of RL, and Group B received 15 ml/kg of RL, both administered over 30 minutes before anesthesia induction. Hemodynamic parameters, including heart rate (HR), systolic blood pressure (SBP), diastolic blood pressure (DBP), and mean arterial pressure (MAP), were monitored at predefined intervals following induction of anesthesia. Hypotension was defined as a >30% fall in MAP from baseline or MAP < 60 mmHg, with mephentermine administered as needed. Statistical analysis used t-tests, Mann-Whitney U-tests, and chi-square tests.

Results

The 88 participants were demographically comparable across both groups. While the mean inferior vena cava (IVC) maximum values were similar, Group B showed a significantly higher mean IVCCI (48.1% vs. 43%; p < 0.001) compared to Group A, despite receiving more fluid. Post-induction, heart rate remained comparable. However, Group B consistently maintained significantly higher SBP, DBP, and MAP at multiple time points compared to Group A. Notably, four patients in Group A (10 ml/kg RL), all with pre-existing hypertension, required mephentermine for a >30% fall in SBP, whereas no patients in Group B (15 ml/kg RL) required vasopressor intervention.

Conclusion

The study reinforces the challenge of PIH and the utility of IVC ultrasound in assessing fluid status. It found that a higher dose of RL (15 ml/kg) was more effective in preventing PIH than 10 ml/kg, as evidenced by better-maintained SBP, DBP, and MAP and the complete absence of vasopressor requirements in the higher-dose group. The significant IVCCI difference between groups, despite higher fluid in Group B, suggests a greater baseline fluid responsiveness in this group. These findings suggest that 15 ml/kg of pre-induction RL, guided by IVCCI, improves hemodynamic stability, particularly beneficial for hypertensive patients, advocating for an individualized, dynamic fluid management strategy.

## Introduction

The effect of hemodynamic variation during anaesthesia induction is critical for preventing adverse postoperative outcomes. Hypotension after induction is common [1], with incidence ranging from 5% to 99% [2]. Factors predisposing to post-induction hypotension (PIH) include American Society of Anesthesiologists (ASA) physical status, low baseline mean arterial pressure (MAP), increased age, and the use of induction agents like propofol and high-dose opioids [3]. A frequently overlooked yet crucial factor is the patient's preoperative volume status, which varies based on physical condition, bowel preparation, nil per oral (NPO) status, and comorbidities.

Intraoperative hypotension (IOH) severely impacts major organ systems, elevating the perioperative risk of myocardial injury, acute kidney injury, stroke, and septic complications. Studies [4] have shown that even short durations of MAP <80 mmHg, or any exposure to MAP <55 mmHg, are linked to end-organ damage. IOH also increases postoperative morbidity, mortality, and the length of ICU or hospital stays [5].

Therefore, preoperative assessment of volume status before general anesthesia (GA) is paramount. Traditional monitoring methods like blood pressure, heart rate (HR), and non-invasive cardiac output monitors often fail to reliably identify fluid responders [6]. Similarly, invasive methods such as pulmonary arterial catheters and central venous pressure measurements are static preload indicators with poor predictive value and inherent limitations [6].

A simple, non-invasive alternative is ultrasonography, which can precisely identify hypovolemia and guide volume optimization before GA, thereby preventing PIH [7]. Ultrasound-guided measurement of the inferior vena cava (IVC) diameter, particularly the Inferior Vena Cava Collapsibility Index (IVCCI), has proven effective in predicting fluid responsiveness in both mechanically ventilated and spontaneously breathing patients [8-9]. In spontaneously breathing patients, IVCCI shows 71% sensitivity and 81% specificity for predicting hypotension in volume-depleted cases [8-9].

While IVCCI-guided volume loading is well-studied for preventing spinal anesthesia-induced hypotension, its application in GA patients is limited and shows varying results due to diverse patient populations and hypotension definitions. Studies suggest Ringer’s lactate (RL) at doses like 10 ml/kg can prevent PIH, with some indicating co-loading reduces vasopressor requirements compared to preloading [10]. However, the optimal dose remains controversial; a meta-analysis found no significant difference between preloaded and co-loaded patients with 15 mL/kg crystalloids, and some research suggests avoiding higher volumes (e.g., 20 mL/kg vs 10 mL/kg showing similar results) [11-12].

Given the lack of clear guidelines, this study aims to determine the optimum dose of intravenous RL to prevent PIH by measuring maximum and minimum IVC diameters (dIVCmax, dIVCmin), IVC-CI, and basal and post-induction MAP.

## Materials and methods

Study design

This was a prospective, randomized, interventional study. This study included patients between 18 and 65 years with ASA physical status I or II, baseline IVCCI > 40%, undergoing elective surgery under GA. Whereas patients with systolic blood pressure ≥ 180 mmHg or < 90 mmHg, pregnant women, patients with abdominal mass or ascites, emergency surgery, and patients in whom a good sonological window could not be obtained were excluded from the study.

Ethical approval

Approval by the institutional ethics committee of Tata Main Hospital, Jamshedpur, India, was taken (approval number: TMH/IEC/JUNE/011/2022), and written informed consent of all patients was obtained to conduct the study.

Sample size calculation

The sample size was calculated using a standard formula.

$n=\frac{2\sigma^{2}\times(Z_{1-\alpha/2}+Z_{1-\beta})^{2}}{\delta^{2}}$

where n = Sample size, Z 1-α/2 = Value of the standard normal deviate at 95% confidence interval, Z 1-β= Value of the standard normal deviate at 80% power of the study, σ = Pooled standard deviation, and δ = Minimal clinically important difference in blood pressure

A sample size of 80 was calculated using the above formula. However, 88 patients were ultimately enrolled (44 per group) to account for a 10% dropout rate. A sealed opaque envelope randomization technique was used for allocation concealment.

Methodology

The methodology involved preoperative evaluation, fasting, and baseline vital monitoring. IVC ultrasonography was performed by a trained investigator to measure dIVCmax and dIVCmin. The IVCCI was calculated as follows:

$\frac{(\mathrm{IVC}_{\max}-\mathrm{IVC}_{\min})}{\mathrm{IVC}_{\max}}\times 100$

Patients with IVCCI ≥ 40% were randomized to receive either 10ml/kg (Group A) or 15ml/kg (Group B) of RL over 30 minutes prior to anesthesia induction. Standard institutional anesthesia induction was followed. Vital parameters like HR, oxygen saturation (SpO₂), systolic blood pressure (SBP), diastolic blood pressure (DBP), and MAP were noted at predefined intervals following induction. Hypotension, defined as a >30% fall in SBP from baseline or MAP < 60 mmHg, was treated with intravenous mephentermine.

Primary outcomes included incidence of hypotension and changes in HR, SBP, DBP, MAP, and SpO₂ at specific time points, while secondary outcomes analyzed vasoactive drug and IV fluid administration.

Data collected were age, sex, height, weight, body mass index (BMI), ASA grade, type of surgery, and patients' history of preexisting comorbidities, along with the hemodynamic parameters as mentioned above.

Statistical analysis

Data recording was done using Microsoft Excel (Microsoft Corp., Redmond, WA, USA), and statistical analysis was performed with IBM SPSS Statistics software for Windows, version 28.0. Continuous variables were presented as mean ± SD or median (min - max), and categorical variables were presented as absolute numbers and percentages. Data were checked for normality before statistical analysis. Normally distributed continuous variables were compared using the unpaired t-test, whereas the Mann-Whitney U test was used for those variables that were not normally distributed. Categorical variables were analyzed using either the chi-square test or Fisher’s exact test as appropriate. P-value <0.05 was considered significant.

## Results

The study comprised 88 participants with a mean age of 49.9 ± 10.8 years, ranging from 24 to 65 years. Demographic analysis confirmed that the cohorts were well-matched, with no statistically significant difference in age distribution between Group A (51.5 ± 10.0 years) and Group B (46.8 ± 11.7 years; p=0.133). Gender representation was similarly balanced across the population (44.4% female; 55.6% male), with both groups exhibiting statistically comparable distributions (p=0.497).

Anthropometric assessments, including weight, height, and BMI, remained consistent across both study arms. Group A and Group B showed comparable mean weights (62.9 kg vs. 65.9 kg; p=0.272) and heights (158.4 cm vs. 160.8 cm; p=0.202). Consequently, the mean BMI values (25.0 kg/m² vs. 25.4 kg/m²; p=0.578) indicated a uniform physical profile across the participant base, ensuring baseline parity for these physical parameters.

The clinical profile of the participants was characterized by a 32.1% prevalence of hypertension and a 12.3% prevalence of diabetes mellitus, with other conditions accounting for 16.0% of the population. Assessment of physical status using ASA grading further confirmed group consistency, with no significant difference in the distribution of Grade I and Grade II classifications (p=0.963). Specifically, Grade II status was observed in 77.3% of Group A and 75% of Group B, reflecting a comparable baseline health status between the two cohorts.

IVC parameters and IVCCI

Analysis of the IVC dimensions revealed that while mean maximum values were identical across both cohorts (1.5 ± 0.3 cm; p=0.424), a statistically significant divergence was observed in the mean minimum values. Specifically, Group B recorded a lower mean dIVCmin of 0.8 ± 0.2 cm compared to 0.9 ± 0.2 cm in Group A (p=0.040).

This variation in minimum diameter underpinned a significantly higher mean IVCCI in Group B (48.1 ± 6.6%) relative to Group A (43 ± 7.7%; p < 0.001). Crucially, despite receiving a larger fluid bolus of 15 ml/kg RL, the elevated CI in Group B indicates a more pronounced volume deficit or a higher degree of fluid responsiveness compared to the 10 ml/kg RL cohort.

Hemodynamic parameters post induction

*Heart Ra*te

**Figure 1:**
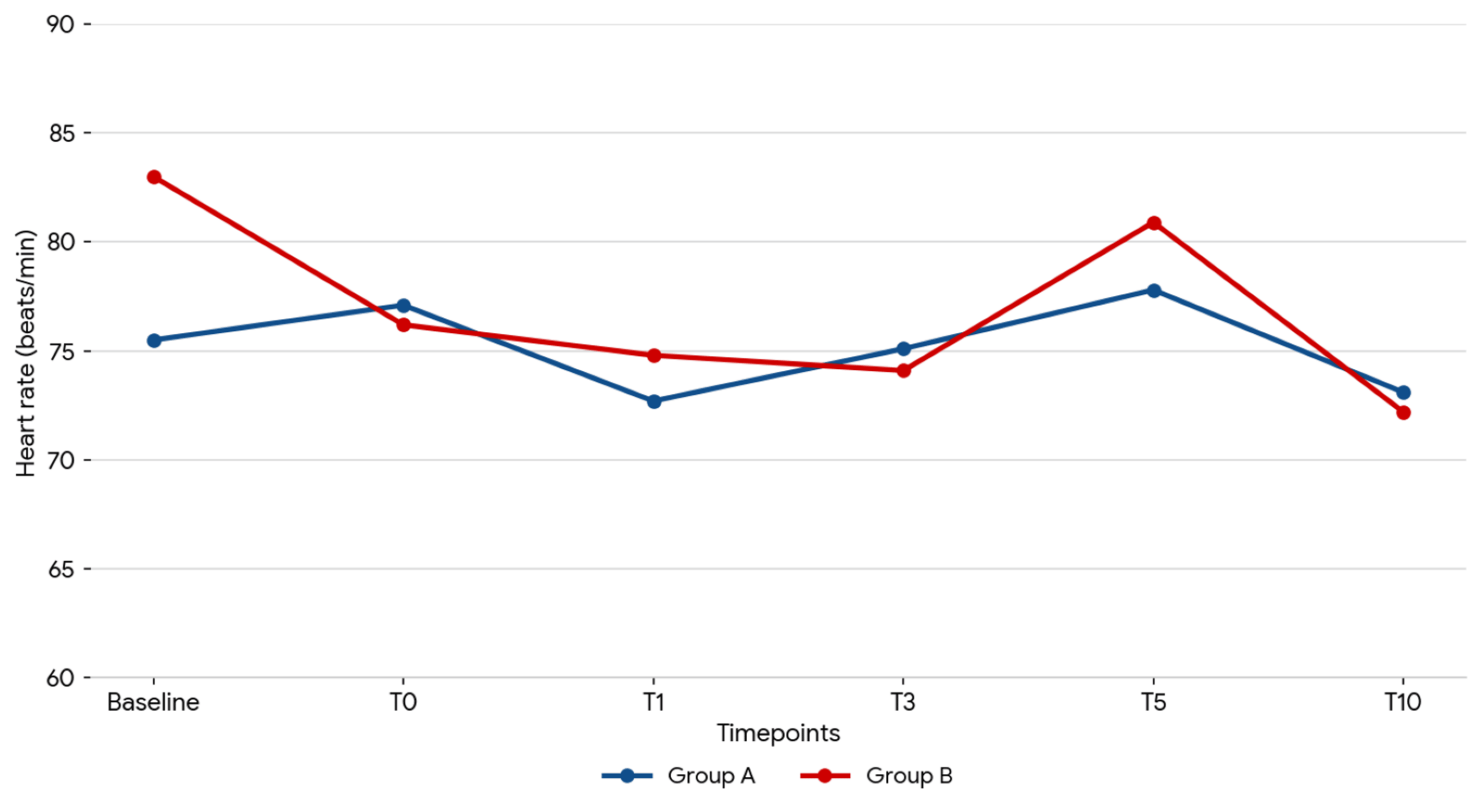
Comparison of heart rate at different time points between the two groups

No statistically significant differences in HR were observed between Group A and Group B (Figure 1) at baseline or at any time point post induction (0, 1, 3, 5, and 10 minutes being marked as T0, T1, T3, T5, T10).

Systolic Blood Pressure

**Figure 2:**
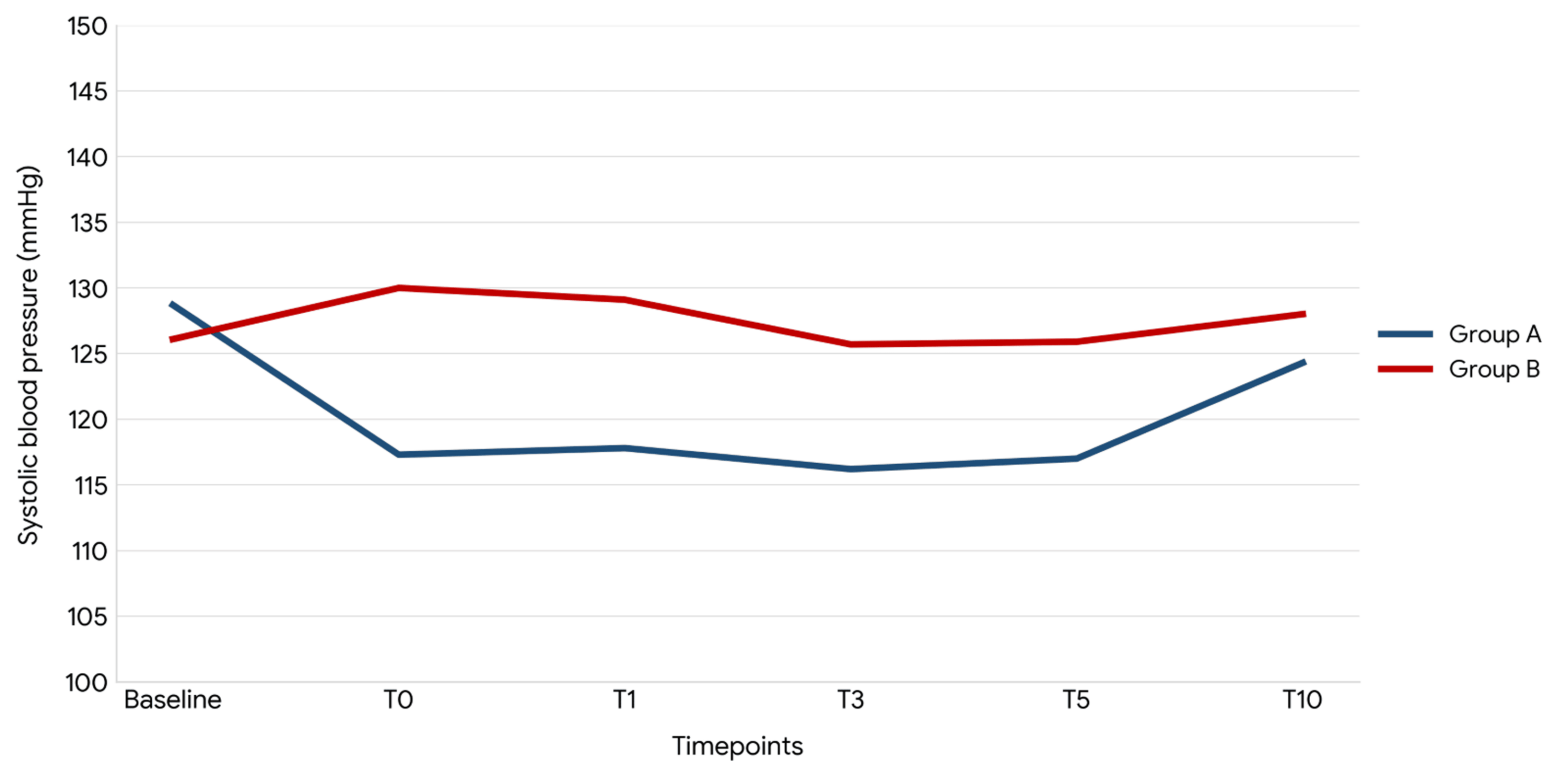
Comparison of systolic blood pressure at different time points between the two groups

While baseline SBP was comparable (p=0.518), statistically significant differences were found in SBP at 0, 1, 3, and 5 minutes post induction, with Group B showing higher SBP values (Figure 2). No significant difference was noted at 10 minutes (p=0.101).

Diastolic Blood Pressure

**Figure 3:**
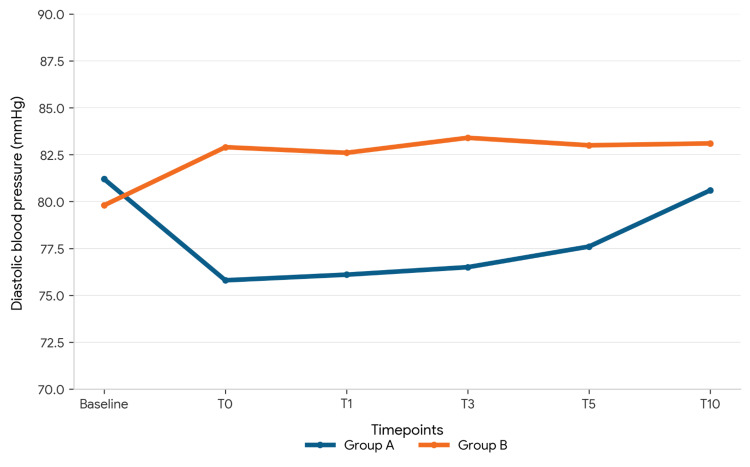
Comparison of diastolic blood pressure at different time points between the two groups

Baseline DBP was similar (p=0.817). However, statistically significant differences in mean DBP were observed at all post-induction time points (0, 1, 3, 5, and 10 minutes), with Group B maintaining higher DBP (Figure 3).

Mean Arterial Pressure

**Figure 4:**
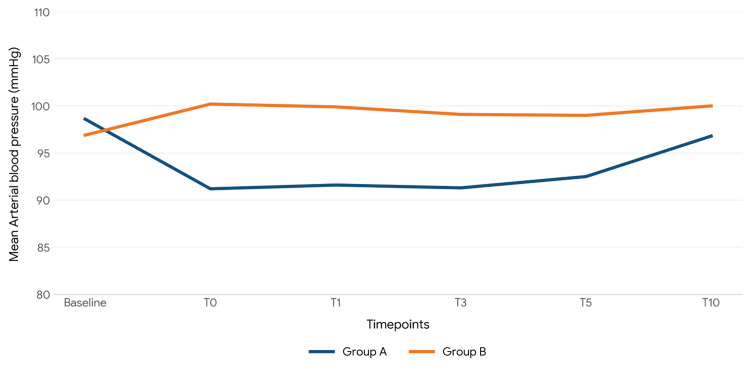
Comparison of mean arterial blood pressure at different time points between the two groups

Baseline MAP was comparable (p=0.496). However, statistically significant differences were found in MAP at 0, 1, 3, 5, and 10 minutes post induction, with Group B consistently demonstrating higher MAP values (Figure 4).

Incidence of hypotension and mephentermine use

Twenty-two patients in Group B were hypertensives, but none of them developed hypotension and therefore, none of them required mephentermine. Four out of 21 hypertensive patients of Group A developed hypotension, which was treated with mephentermine. This only shows that 20ml/kg RL (Group B) was more effective in the prevention of PIH as compared to 10ml/kg (Group A).

## Discussion

Hypotension during anesthesia is a frequent occurrence, linked to increased perioperative morbidity and mortality. Its definition varies widely in literature, ranging from MAP < 65 mmHg or a >30% fall in MAP [13], to SBP < 90 mmHg or a ≥30% fall in SBP [14]. This variability leads to reported post-induction hypotension rates from 5% to 99% [2]. In our study, hypotension was defined as MAP < 60 mmHg or a >30% fall in SBP from baseline, consistent with Zhang et al. [7].

Multiple factors contribute to IOH, including advanced age, anesthetic induction agents like propofol, and high-dose opioids [1,3]. Patient preoperative volume status is another critical, often overlooked, predictor, influenced by physical condition, bowel preparation, fasting, and comorbidities [15]. Volume optimization is crucial, but accurate assessment is challenging. While various invasive static and dynamic methods exist, including central venous pressure, pulmonary capillary wedge pressure, stroke volume variation, pulse pressure variation, transesophageal echocardiography, and esophageal Doppler ultrasound [16,17], they are often time-consuming, require specialized equipment, and are not routinely feasible.

Point-of-care ultrasound, specifically ultrasound-guided IVC parameters, has emerged as a promising non-invasive bedside tool for assessing volume status and predicting fluid responsiveness in emergency, intensive care, and operating room settings [17-19].

This study aimed to determine the optimal dose of RL to prevent PIH in GA patients by assessing the IVCCI. Eighty-eight elective surgery patients under GA were included. dIVCmax and dIVCmin were measured to calculate IVCCI.

Previous studies by Zhang et al. [7] and Muller et al. [20] found IVCCI cutoff values of 43% and 40%, respectively, with good sensitivity and specificity for predicting hypotension. While a universal cutoff of 50% predicts fluid responsiveness, Szabó et al. [14] noted its low sensitivity (45.5%) despite high specificity (90%), suggesting a 55% chance of missing cases prone to hypotension. Therefore, our study adopted a 40% IVCCI cutoff.

RL, an isotonic solution, was chosen for its ability to expand intravascular volume, increase preload, improve tissue perfusion, and provide bioenergetic fuel [21]. Fluid loading has been extensively studied for preventing post-spinal hypotension, comparing RL with colloids or different RL doses. One study suggested similar efficacy between 10 ml/kg and 20 ml/kg of RL for preventing post-spinal hypotension, recommending against larger volumes to avoid complications [12,22,23]. Colloids are often avoided due to cost, allergic reactions, and potential coagulation effects.

Despite numerous studies on preventing PIH, research on preoperative crystalloid use to prevent PIH is scarce. Pradhan et al. [24] found that preloading with RL 12 ml/kg and pre-induction intravenous ephedrine 70 mcg/kg were equally effective against propofol-induced hypotension, though ephedrine caused higher heart rate variability. Agarwal et al. [10] observed that 10 ml/kg RL successfully counteracted propofol-induced hypotension, unlike ephedrine. Paul et al. [25] demonstrated that preanesthetic fluid loading with RL (20 mL/kg) reduced PIH during cervical spine surgery.

Given the lack of explicit guidelines on optimal preoperative RL dosage, our study sought to determine this. We initially calculated IVCCI and then randomized 88 patients into two groups (n=44 each): Group A received RL 10 ml/kg, and Group B received RL 15 ml/kg, both administered over 30 minutes before induction. Both groups were comparable in demographics (age, gender, anthropometric characteristics), ASA grading, and comorbidities.

After RL preloading, patients were shifted to the operating theater, and baseline vitals were recorded. Post induction, HR, SBP, DBP, and MAP were monitored at 0, 1, 3, 5, and 10 minutes, avoiding hemodynamic fluctuations from laryngoscopy/intubation. Baseline parameters were comparable.

HR showed no statistically significant differences between groups at any time point. However, SBP, DBP, and MAP showed statistically significant differences between Group A and Group B at 0, 1, 3, and 5 minutes post induction, with SBP showing no significant difference at 10 minutes.

The mean MAP in our study remained above 60 mmHg at all observed time points. Four patients in Group A experienced a >30% fall in SBP from baseline, requiring a single 6 mg bolus of mephentermine injection. Notably, no participants in Group B (15 ml/kg RL) required mephentermine. The four patients in Group A who needed mephentermine were hypertensive, suggesting a higher propensity for hemodynamic instability.

Limitations of the study

The study was conducted in a single center. Blinding was not possible. Only ASA 1 and 2 patients were included in this study. The change in IVC parameters was not measured after preloading with RL, and the sample size was small.

## Conclusions

This study suggests that a higher dose of RL (15 ml/kg) effectively reduces the incidence of PIH, as evidenced by better maintenance of SBP, DBP, and MAP, and a complete absence of vasopressor requirement, compared to a 10 ml/kg dose. The significant difference in IVCCI between the groups indicates a potential role for IVCCI in predicting fluid responsiveness and guiding fluid management strategies to prevent post-induction hypotension.

The findings highlight that an individualized approach, potentially guided by dynamic fluid assessment, is crucial for optimal hemodynamic stability during anesthesia induction, especially in patients with pre-existing conditions like hypertension.
